# Clinical Trial Evidence Supporting US Food and Drug Administration Approval of Novel Cancer Therapies Between 2000 and 2016

**DOI:** 10.1001/jamanetworkopen.2020.24406

**Published:** 2020-11-10

**Authors:** Aviv Ladanie, Andreas M. Schmitt, Benjamin Speich, Florian Naudet, Arnav Agarwal, Tiago V. Pereira, Francesco Sclafani, Amanda K. Herbrand, Matthias Briel, Juan Martin-Liberal, Thomas Schmid, Hannah Ewald, John P. A. Ioannidis, Heiner C. Bucher, Benjamin Kasenda, Lars G. Hemkens

**Affiliations:** 1 Department of Clinical Research, Basel Institute for Clinical Epidemiology and Biostatistics, University Hospital and University of Basel, Basel, Switzerland; 2 Swiss Tropical and Public Health Institute, Basel, Switzerland; 3 Medical Oncology, University Hospital and University of Basel, Basel, Switzerland; 4 Centre for Statistics in Medicine, Nuffield Department of Orthopaedics, Rheumatology and Musculoskeletal Sciences, University of Oxford, Oxford, United Kingdom; 5 Universite de Rennes, CHU Rennes, Inserm, CIC 1414–Centre d’Investigation Clinique de Rennes, Rennes, France; 6 Department of Medicine, University of Toronto, Toronto, Ontario, Canada; 7 Department of Health Research Methods, Evidence and Impact, McMaster University, Hamilton, Ontario, Canada; 8 Applied Health Research Centre, Li Ka Shing Knowledge Institute, St Michael's Hospital, Toronto, Ontario, Canada; 9 Department of Health Sciences, College of Medicine, University of Leicester, Leicester, United Kingdom; 10 Department of Medical Oncology, Institut Jules Bordet, Brussels, Belgium; 11 St Clara Hospital, Basel, Switzerland; 12 Melanoma, Sarcoma and GU Tumors Unit, Catalan Institute of Oncology Hospitalet, Barcelona, Spain; 13 University Medical Library, University of Basel, Basel, Switzerland; 14 Meta-Research Innovation Center at Stanford, Stanford University, Stanford, California; 15 Department of Medicine, Stanford University School of Medicine, Stanford, California; 16 Department of Epidemiology and Population Health, Stanford University School of Medicine, Stanford, California; 17 Department of Biomedical Data Science, Stanford University School of Medicine, Stanford, California; 18 Department of Statistics, Stanford University School of Humanities and Sciences, Stanford, California

## Abstract

**Importance:**

Clinical trial evidence used to support drug approval is typically the only information on benefits and harms that patients and clinicians can use for decision-making when novel cancer therapies become available. Various evaluations have raised concern about the uncertainty surrounding these data, and a systematic investigation of the available information on treatment outcomes for cancer drugs approved by the US Food and Drug Administration (FDA) is warranted.

**Objective:**

To describe the clinical trial data available on treatment outcomes at the time of FDA approval of all novel cancer drugs approved for the first time between 2000 and 2016.

**Design, Setting, and Participants:**

This comparative effectiveness study analyzed randomized clinical trials and single-arm clinical trials of novel drugs approved for the first time to treat any type of cancer. Approval packages were obtained from drugs@FDA, a publicly available database containing information on drug and biologic products approved for human use in the US. Data from January 2000 to December 2016 were included in this study.

**Main Outcomes and Measures:**

Regulatory and clinical trial characteristics were described. For randomized clinical trials, summary treatment outcomes for overall survival, progression-free survival, and tumor response across all therapies were calculated, and median absolute survival increases were estimated. Tumor types and regulatory characteristics were assessed separately.

**Results:**

Between 2000 and 2016, 92 novel cancer drugs were approved by the FDA for 100 indications based on data from 127 clinical trials. The 127 clinical trials included a median of 191 participants (interquartile range [IQR], 106-448 participants). Overall, 65 clinical trials (51.2%) were randomized, and 95 clinical trials (74.8%) were open label. Of 100 indications, 44 indications underwent accelerated approval, 42 indications were for hematological cancers, and 58 indications were for solid tumors. Novel drugs had mean hazard ratios of 0.77 (95% CI, 0.73-0.81; *I*^2^ = 46%) for overall survival and 0.52 (95% CI, 0.47-0.57; *I*^2^ = 88%) for progression-free survival. The median tumor response, expressed as relative risk, was 2.37 (95% CI, 2.00-2.80; *I*^2^ = 91%). The median absolute survival benefit was 2.40 months (IQR, 1.25-3.89 months).

**Conclusions and Relevance:**

In this study, data available at the time of FDA drug approval indicated that novel cancer therapies were associated with substantial tumor responses but with prolonging median overall survival by only 2.40 months. Approval data from 17 years of clinical trials suggested that patients and clinicians typically had limited information available regarding the benefits of novel cancer treatments at market entry.

## Key Points

**Question:**

What are the available data on cancer treatment outcomes for new cancer therapies approved by the US Food and Drug Administration?

**Findings:**

In this comparative effectiveness study of 92 novel cancer therapies approved for 100 indications over 17 years, 44% of drug approvals were based on data from nonrandomized clinical trials. Randomized clinical trials typically reported that these drugs were associated with substantial tumor responses and delays in the time to progression or death, but the median absolute increase in overall survival was only 2 months.

**Meaning:**

This study’s findings indicate that, at the time of drug approval, limited supporting data are available to decision-makers, and the increase in overall survival associated with new cancer drugs is typically small.

## Teaser

This comparative effectiveness research examines clinical trial data on treatment outcomes used to support US Food and Drug Administration approval of novel cancer therapies that were approved for the first time between 2000 and 2016.

## Introduction

Cancer research is characterized by the perceived urgency to develop novel drugs that may improve patients’ survival and quality of life. Before patients have access to novel therapies, the available evidence on benefits and harms from clinical trials is assessed by authoritative institutions, such as the US Food and Drug Administration (FDA). Several regulatory programs have been established to expedite the development and approval of drugs for serious conditions, such as cancer.^1^ These programs may allow patients to have earlier access to beneficial drugs; however, there is concern that these programs may increase uncertainty in clinical decision-making, as approvals based on these regulations often rely on evidence from fewer and smaller studies, surrogate outcomes, and studies that are more likely to be biased owing to a lack of randomization and adequate controls.^2,3,4,5,6^

Previous analyses have described the evidence used to support FDA approval of cancer therapies for periods before 2013,^4,7,8,9,10^ with a focus on certain types of cancer^11,12,13,14,15^ or on the use of certain end points in clinical trials aimed at drug approval.^6,10^ The objective of our study was to systematically investigate the available data on treatment outcomes for all cancer drugs approved by the FDA for the first time between 2000 and 2016. We described the regulatory characteristics and supporting clinical trials and calculated the treatment outcomes of overall survival (OS), progression-free survival (PFS), and tumor response.

## Methods

### Database

This comparative effectiveness study was performed as part of the Comparative Effectiveness of Innovative Treatments in Cancer (CEIT-Cancer) project. Full details regarding the database and the processes used for data identification, selection, extraction, and handling have been described elsewhere.^16,17^ This study followed the Strengthening the Reporting of Observational Studies in Epidemiology (STROBE) reporting guideline when applicable.^18^ We used only published information and aggregated clinical trial–level data. The University of Basel and University Hospital Basel, Switzerland, do not require institutional review board approval for this type of research as the data used were anonymized and not subject to the health regulations of Switzerland (as confirmed by the regulatory team at the Department of Clinical Research, University of Basel).

In brief, we identified all novel drugs and therapeutic biologic therapies (ie, new molecular entities or novel biologic drugs approved for the first indication) that received FDA approval as treatment for any malignant disease for the first time between January 2000 and December 2016. In this study, we did not consider any additional indications for drugs that received later approval for those indications. We excluded supportive care or imaging drugs that did not produce anticancer activity. We obtained the corresponding FDA approval documents from drugs@FDA,^19^ a publicly available database containing information on drug and biologic products approved for human use in the US, and we searched the documents for randomized clinical trials (RCTs) in which the novel drug was compared with some type of active control, placebo, or standard of care and for single-arm clinical trials that may have provided data on treatment benefits. Dose-comparison clinical trials, in which all patients received the novel drug at different doses without the use of any control arm, were considered together with the single-arm clinical trials, merging all doses. We included all RCTs that were explicitly labeled as pivotal and any other RCT that was conducted in the target population and that compared the novel drug with a control that did not contain the novel drug.

In addition, we included single-arm clinical trials that were explicitly described as pivotal or that we inferred were pivotal and essential for drug approval (eg, based on statements such as, “The clinical review of efficacy was primarily based on an analysis of clinical trial 101-09”).^20^ We extracted characteristics of the drugs, indications, clinical and regulatory details, and features of all eligible clinical trials. For RCTs, we extracted the reported treatment outcomes for OS, PFS, and tumor response. All steps and extractions were conducted by 2 independent reviewers (A.L. and either A.A., B.K., B.S., F.N., F.S., H.E., J.M.-L., T.S., or T.V.P.; A.L. and A.K.H. for extractions of line of treatment, type of control, and approval pathway). Any disagreement was resolved by consensus or by a third reviewer (A.M.S. or B.K.). Only information on sample size, clinical trial phase, and blinding was extracted by 1 reviewer (A.L.) alone.

### Statistical Analysis

We used descriptive statistics to analyze drugs, indications, clinical and regulatory details, clinical trial characteristics, and treatment outcomes. We used only RCTs for the analysis of treatment outcomes because single-arm clinical trials and dose-comparison clinical trials do not provide comparative treatment outcomes for experimental drugs. In five 3-arm RCTs that evaluated the experimental treatment using 2 different doses, we selected the comparison with the later-approved dose. Three studies were considered twice because each was pertinent to 2 indications for the same drug.

We combined treatment outcomes from all RCTs in meta-analyses using random-effects models.^21^ We described the statistical heterogeneity using the *I*^2^ statistic.^22^ Tumor response rates were presented as unadjusted relative risks (RRs). Odds ratios (ORs) were also reported to address potential differences between measures when events were frequent or rare. We used a continuity correction of 0.5 to account for cases of 0 events. The increase in OS and PFS per study was calculated as the difference between the median OS or PFS of the experimental vs control arms for all indications for which data on the median OS or PFS were available.

Analyses were conducted overall and stratified by cancer type (solid tumors vs hematological cancers), orphan status (with vs without; based on the Orphan Drug Act,^23^ which provides incentives for pharmaceutical manufacturers to develop drugs to treat rare diseases), and approval pathway (nonaccelerated vs accelerated; accelerated pathway based on the FDA Accelerated Approval Program,^24^ which enables earlier approval of drugs that treat serious diseases and address an unmet medical need). We compared the OS and PFS effect sizes by calculating the ratio of hazard ratios (HRs). We also conducted several sensitivity and subset analyses (eTable 1 and eTable 2 in the Supplement).

All analyses were exploratory. Data were analyzed using Microsoft Excel (Microsoft Corp); R software, version 3.5.1 (R Foundation for Statistical Computing); RStudio software, version 1.1.383 (RStudio, PBC); and Stata software, version 14.2 (StataCorp LLC).

## Results

We identified 92 novel cancer drugs approved between January 2000 and December 2016 for 100 indications (7 drugs with multiple indications) (Table 1). A total of 42 indications (42.0%) were for hematological cancers, and 58 indications (58.0%) were for solid tumors. Most drugs were first-line (30 drugs [30.0%]) and second-line (51 drugs [51.0%]) treatments, and only 19 drugs (19.0%) were third- or fourth-line treatments. For 28 drugs (28.0%), FDA approval was subject to the confirmatory testing of a specific biomarker (eg, a certain tumor variant). The accelerated approval program was used for 44 indications (44.0%), and 66 indications (66.0%) had orphan status. All hematological treatments had orphan status (eFigure 1 in the Supplement).

**Table 1.** Treatment Indication Characteristics

Treatment	Indications, No.
Total	100
Cancer type	
Solid tumors	58
Genitourinary	14
Gastrointestinal	9
Respiratory and thoracic	9
Skin	9
Breast	8
Endocrine and neuroendocrine	3
Gynecological	2
Sarcoma and GIST	3
Neurological	1
Hematological cancers	42
Leukemia	7
Lymphoma^a^	18
Multiple myeloma	7
Chronic myelogenous leukemia	6
Other^b^	4
Line of treatment	
1	30
2	51
≥3	19
Biomarker	28
*BCR-ABL* rearrangement	6
*BRAF* variant	4
*ERBB2* (formerly *HER2*) positivity	3
*EGFR* expression	3
*ALK* variant	3
*BRCA* variant	2
Other	6
Regulatory approval designation	
Priority review^c^	77
Orphan status	66
Accelerated approval	44
Breakthrough therapy^d^	15

Abbreviations: *ALK*, anaplastic lymphoma kinase gene; *BCR-ABL*, fusion gene of breakpoint cluster region gene and *ABL1* protooncogene; *BRAF*, v-Raf murine sarcoma viral oncogene homologue B1 gene; *BRCA*, breast cancer gene; *EGFR*, epidermal growth factor receptor gene; GIST, gastrointestinal stromal tumor; *HER2*, human epidermal growth factor receptor 2 gene.

^a^ Includes chronic lymphatic leukemia.

^b^ Includes myelodysplastic syndromes (n = 2), myelofibrosis, and multicentric Castleman disease.

^c^ Priority review designation status of 4 indications could not be ascertained.

^d^ Approvals in 2013 and later.

### Characteristics of Clinical Trials

We included 127 clinical trials with a median of 191 enrolled participants (interquartile range [IQR], 106-448 participants) (Table 2). A total of 65 clinical trials (51.2%) had less than 200 participants. The median number of eligible clinical trials per approved indication was 1 (range, 1-5). For 76 of 100 indications, only 1 clinical trial was eligible (45 RCTs and 31 single-arm clinical trials). For 20 of 100 indications, 2 clinical trials (8 indications with 2 RCTs, 11 indications with 2 single-arm clinical trials, 1 indication with 1 RCT, and 1 single-arm clinical trial) were eligible, and for 2 of 100 indications, 3 clinical trials were eligible (1 indication with 3 single-arm clinical trials, 1 indication with 2 RCTs, and 1 single-arm clinical trial). A total of 44 of 100 indications received FDA approval without supporting evidence from RCTs. Clinical trials conducted to support solid tumor (median, 330 participants; interquartile range [IQR], 171-638 participants), nonorphan (median, 435 participants; IQR, 230-760 participants), and nonaccelerated approval (median, 374 participants; IQR, 159-710 participants) indications were larger than those conducted to support hematological cancer (median, 111 participants; IQR, 74-188 participants), orphan (median, 152 participants; IQR, 94-292 participants), and accelerated approval (median, 136 participants; IQR, 100-202 participants) indications (Table 2).

**Table 2.** Clinical Trial Characteristics

Characteristic	No. (%)						
	Overall	Cancer type		Orphan status		Accelerated approval	
		Solid tumor	Hematological cancer	No	Yes	No	Yes
Total clinical trials, No.	127	72	55	41	86	66	61
Participants, median (IQR)	191 (106-448)	330 (171-638)	111 (74-188)	435 (230-760)	152 (94-292)	374 (159-710)	136 (100-202)
Study design							
RCT	65 (51.2)	51 (70.8)	14 (25.5)	31 (75.6)	34 (39.5)	54 (81.8)	11 (18.0)
Single-arm clinical trial^a^	62 (48.8)	21 (29.2)	41 (74.5)	10 (24.4)	52 (60.5)	12 (18.2)	50 (82.0)
Clinical trial phase (drug development phase)							
Phase 3	57 (44.9)	44 (61.1)	13 (23.6)	27 (65.9)	30 (34.9)	50 (75.8)	7 (11.5)
Phase 2	66 (52.0)	25 (34.7)	41 (74.5)	14 (34.1)	52 (60.5)	15 (22.7)	51 (83.6)
Phase 1	3 (2.4)	3 (4.2)	0	0	3 (3.5)	0	3 (4.9)
Not reported	1 (0.8)	0	1 (1.8)	0	1 (1.2)	1 (1.5)	0
Type of blinding							
Double	30 (23.6)	25 (34.7)	5 (9.1)	16 (39.0)	14 (16.3)	27 (40.9)	3 (4.9)
Single	1 (0.8)	1 (1.4)	0	0	1 (1.2)	1 (1.5)	0
Open label	95 (74.8)	45 (62.5)	50 (90.9)	25 (61.0)	70 (81.4)	37 (56.1)	58 (95.1)
Not specified	1 (0.8)	1 (1.4)	0	0	1 (1.2)	1 (1.5)	0
Type of control							
Parallel without experimental drug	65 (51.2)	51 (70.8)	14 (25.5)	31 (75.6)	34 (39.5)	54 (81.8)	11 (18.0)
Randomized							
Active^b^	23 (18.1)	19 (26.4)	4 (7.3)	11 (26.8)	12 (14.0)	17 (25.8)	6 (9.8)
Placebo	26 (20.5)	22 (30.6)	5 (9.1)	12 (29.3)	14 (16.3)	24 (36.4)	2 (3.3)
No treatment^c^	16 (12.6)	11 (15.3)	5 (9.1)	7 (17.1)	8 (9.3)	13 (19.7)	3 (4.9)
No or other controls	62 (48.8)	21 (29.2)	41 (74.5)	10 (24.4)	52 (60.5)	12 (18.2)	50 (82.0)
Randomized dose comparison	6 (4.7)	3 (4.2)	3 (5.5)	2 (4.9)	4 (4.7)	1 (1.5)	5 (8.2)
Nonrandomized historical control	56 (44.1)	18 (25.0)	38 (69.1)	8 (19.5)	48 (55.8)	11 (16.7)	45 (73.8)
End point for RCTs							
Total RCTs, No.	65	51	14	31	34	54	11
Overall survival	54 (83.1)	44 (86.3)	10 (71.4)	25 (80.6)	29 (85.3)	48 (88.9)	6 (54.5)
Progression-free survival	54 (83.1)	46 (90.2)	8 (57.1)	26 (83.9)	28 (82.4)	47 (87.0)	7 (63.6)
Tumor response	51 (78.5)	40 (78.4)	11 (78.6)	24 (77.4)	27 (79.4)	45 (83.3)	6 (54.5)

Abbreviations: IQR, interquartile range; RCT, randomized clinical trial.

^a^ Clinical trials without a parallel control (ie, patients were randomized to different doses of the experimental treatment only [dose-comparison clinical trials] or not randomized and compared with historical controls.

^b^ Includes comparators in which placebo is received in addition to active treatment (ie, add-on or double-dummy clinical trials).

^c^ Includes supportive therapy or standard care.

Of 127 clinical trials, 65 studies (51.2%) were RCTs, 66 studies (52.0%) were phase 2 clinical trials, and 30 studies (23.6%) were double-blinded. Of the 62 single-arm clinical trials, 6 studies (9.7%) were dose-comparison clinical trials. Although approvals of treatments for solid tumors (51 of 72 studies [70.8%]), nonorphan indications (31 of 41 studies [75.6%]), and nonaccelerated approval pathways (54 of 66 studies [81.8%]) were typically supported by RCTs, single-arm clinical trials were the most common study design for hematological cancer (41 of 55 studies [74.6%]), orphan (52 of 86 studies [60.5%]), and accelerated approval (50 of 61 studies [82.0%]) indications. The same pattern was observed for clinical trial phase; solid tumor (44 of 72 studies [61.1%]), nonorphan (27 of 41 studies [65.9%]), and nonaccelerated approval (50 of 66 studies [75.8%]) indications were typically supported by phase 3 clinical trials, and hematological cancer (41 of 55 studies [74.6%], orphan (52 of 86 studies [60.5%]), and accelerated approval (51 of 61 studies [83.6%]) indications were more often supported by phase 2 clinical trials. Clinical trials for solid tumor (25 of 72 studies [34.7%]), nonorphan (16 of 41 studies [39.0%]), and nonaccelerated approval (27 of 66 studies [40.9%]) indications were sometimes double-blinded, while double-blinding was rare in clinical trials for hematological cancer (5 of 55 studies [9.1%]), orphan (14 of 86 studies [16.3%]), and accelerated approval (3 of 61 studies [4.9%]) indications.

Overall, 62 of 127 clinical trials (48.8%) had no parallel control without the experimental drug (56 of 127 studies [44.1%] had nonrandomized historical controls, and 6 of 127 studies [4.7%] had randomized dose-comparison controls). Clinical trials supporting the approval of drugs for solid tumors were frequently active-controlled studies (19 of 72 studies [26.4%]), which was also the case for drugs without orphan status (11 of 41 studies [26.8%]) and drugs with nonaccelerated approval (17 of 66 studies [25.8%]). Drugs for hematological cancer (38 of 55 studies [69.1%]), orphan (48 of 86 studies [55.8%]), and accelerated approval (45 of 61 studies [73.8%]) indications typically had no parallel controls.

### Treatment Outcomes

**Figure 1.**
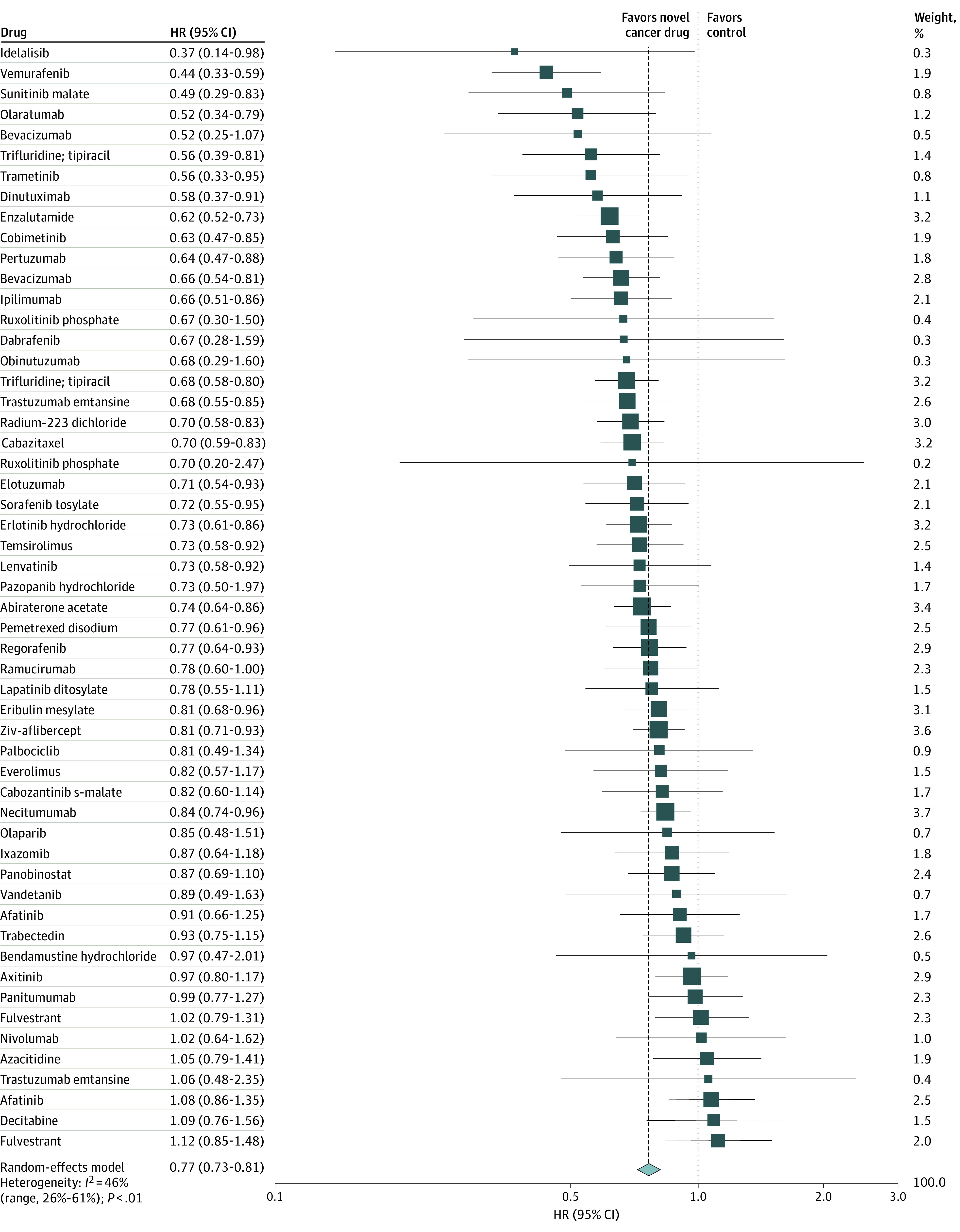
Forest Plot of All Randomized Clinical Trials With Data on Overall Survival Used for Approval of Novel Cancer Drugs Between 2000 and 2016 Squares represent mean values, with the size of the squares indicating weight and horizontal lines representing 95% CIs. Diamonds represent the pooled mean with the points of the diamonds representing 95% CIs. HR indicates hazard ratio.

Across all 54 RCTs with reported treatment outcomes for OS, the combined risk of death associated with any condition across all novel cancer treatments was lower by a mean of 23% compared with the control (HR, 0.77; 95% CI, 0.73-0.81; *I*^2^ = 46%), with a median survival increase of 2.40 months (IQR, 1.25-3.89 months; range, −2.10 to 11.80 months) (Figure 1 and Table 3). The median HR for OS across all studies was 0.74 (IQR, 0.67-0.87; range, 0.37-1.12).

**Table 3.** Treatment Outcomes for Overall Survival, Progression-Free Survival, and Tumor Response

Outcome	Overall		Cancer type				Orphan status				Accelerated approval			
			Solid tumor		Hematological cancer		No		Yes		No		Yes	
	RCTs, No.^a^	Outcome	RCTs, No.	Outcome	RCTs, No.	Outcome	RCTs, No.	Outcome	RCTs, No.	Outcome	RCTs, No.	Outcome	RCTs, No.	Outcome
Overall survival														
HR (95% CI)	54	0.77 (0.73-0.81)^b^	44	0.76 (0.72-0.80)	10	0.86 (0.76-0.98)	25	0.76 (0.71-0.81)	29	0.77 (0.71-0.84)	48	0.76 (0.72-0.80)	6	0.85 (0.71-1.01)
*I*^2^		46		50		2		49		45		47		31
Improvement, median (IQR), mo	35	2.40 (1.25-3.89)	31	2.40 (1.40-3.89)	4	2.15 (0.61-3.58)	21	2.40 (1.44-4.10)	14	2.75 (0.72-3.66)	30	2.20 (1.18-3.66)	5	3.20 (2.70-4.20)
Progression-free survival														
HR (95% CI)	53	0.52 (0.47-0.57)^c^	46	0.53 (0.48-0.58)	7	0.43 (0.28-0.66)	26	0.59 (0.54-0.66)	27	0.44 (0.38-0.52)	46	0.51 (0.46-0.57)	7	0.54 (0.44-0.67)
*I*^2^		88		87		93		84		89		89		66
Improvement, median (IQR), mo	50	2.70 (1.61-4.29)	44	2.40 (1.48-4.22)	6	4.30 (3.95-10.05)	26	2.07 (1.10-4.11)	24	3.50 (2.40-4.64)	43	2.72 (1.68-4.28)	7	2.50 (1.30-5.50)
Tumor response														
RR (95% CI)	50	2.37 (2.00-2.80)	40	2.63 (2.11-3.28)	10	1.81 (1.39-2.35)	24	2.16 (1.71-2.72)	26	2.64 (2.03-3.43)	44	2.49 (2.06-3.00)	6	1.69 (1.10-2.61)
*I*^2^		91		90		93		87		94		92		78
OR (95% CI)	50	3.63 (2.86-4.60)	40	3.54 (2.71-4.61)	10	4.13 (2.30-7.42)	24	2.67 (2.03-3.51)	26	4.75 (3.24-6.97)	44	3.74 (2.90-4.82)	6	2.97 (1.39-6.34)
*I*^2^		85		84		90		78		88		86		74

Abbreviations: HR, hazard ratio; IQR, interquartile range; OR, odds ratio; RCT, randomized clinical trial; RR, relative risk.

^a^ Not all data were reported for all clinical trials.

^b^ Median HR for overall survival, 0.74 (IQR, 0.67-0.87; range, 0.37-1.12).

^c^ Median HR for progression-free survival, 0.55 (IQR, 0.40-0.67; range, 0.16-0.92).

**Figure 2.**
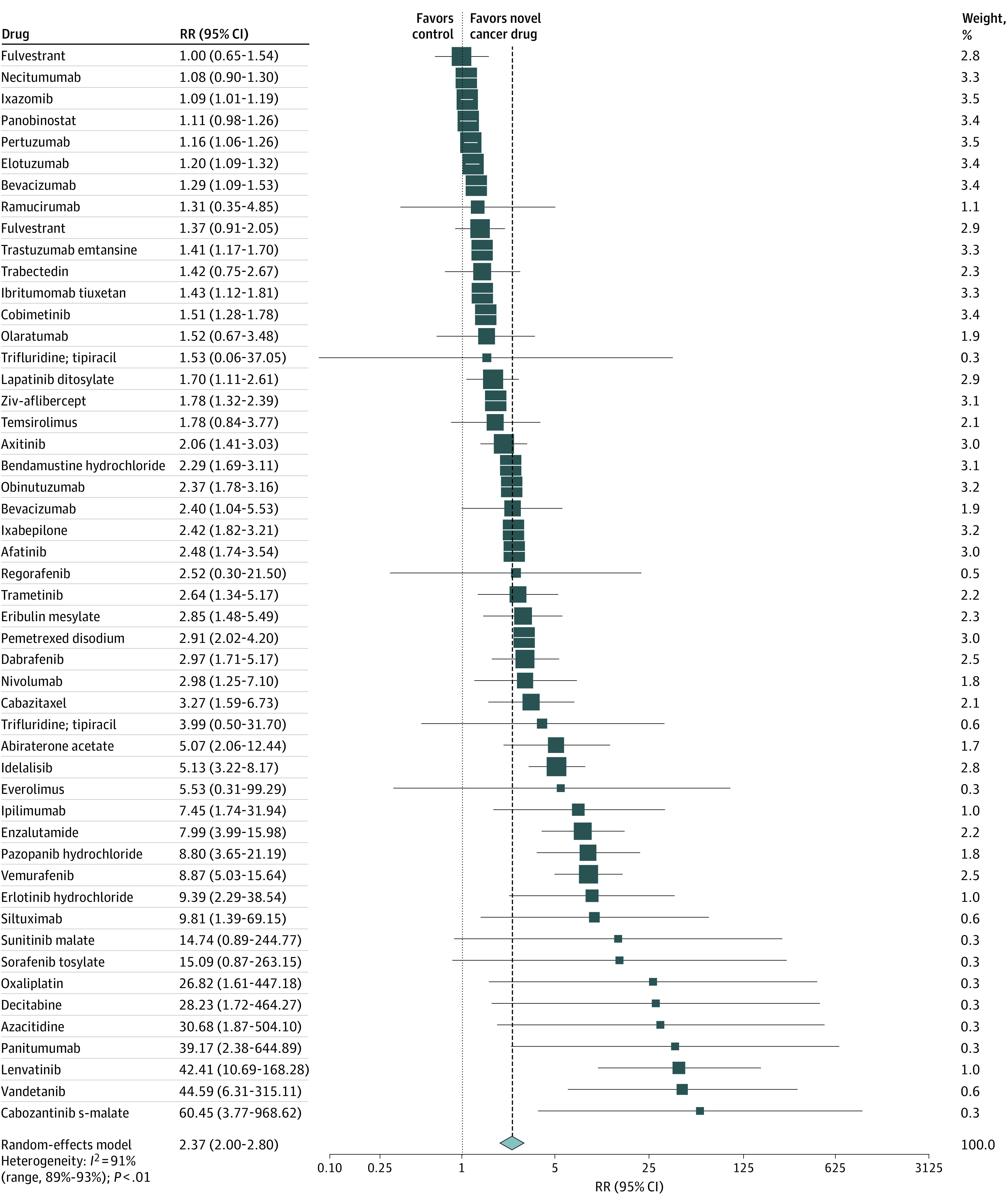
Forest Plot of All Randomized Clinical Trials With Data on Tumor Response Used for Approval of Novel Cancer Drugs Between 2000 and 2016 Squares represent mean values, with the size of the squares indicating weight and horizontal lines representing 95% CIs. Diamonds represent the pooled mean with the points of the diamonds representing 95% CIs. RR indicates relative risk.

The combined risk of tumor progression or death (PFS) associated with any condition was lower by a mean of 48% (HR, 0.52; 95% CI, 0.47-0.57; *I*^2^ = 88%), with a median PFS increase of 2.70 months (IQR, 1.61-4.29 months; range, 0.10 -14.70 months) (Table 3 and eFigure 2 in the Supplement). The median HR for PFS across all studies was 0.55 (IQR, 0.40-0.67; range, 0.16-0.92). Patients who received the novel treatment had a 2.37-fold higher tumor response (95% CI, 2.00-2.80; *I*^2^ = 91%) (Figure 2 and Table 3). The median RR for tumor response across all studies was 2.58 (IQR, 1.51-8.60; range, 1.00-60.45). In 27 of 54 RCTs (50.0%) that reported HRs, the 95% CIs of the HRs for OS were compatible with shorter survival times (ie, values >1.0). This compatibility with shorter PFS times was also observed for PFS in 8 of 54 RCTs (14.8%). In 5 of 54 RCTs (9.3%), the reported 95% CI was compatible with unfavorable outcomes for both PFS and OS.

Treatment outcomes for OS were consistent across subsets for solid tumor (HR, 0.76; 95% CI, 0.72-0.80), orphan (HR, 0.77; 95% CI, 0.71-0.84), nonorphan (HR, 0.76; 95% CI, 0.71-0.81), and full nonaccelerated approval (HR, 0.76; 95% CI, 0.72-0.80) indications (Table 3). Treatment outcomes for hematological cancer (HR, 0.86; 95% CI, 0.76-0.98) and accelerated approval (HR, 0.85; 95% CI, 0.71-1.01) indications were slightly smaller. Median OS increases were similar across all subsets, ranging from 2.15 months (IQR, 0.61-3.58 months) for hematological cancer indications to 3.20 months (IQR, 2.70-4.20 months) for accelerated approval indications. For 31 of the 92 drugs (33.7%), information on absolute OS from RCTs was available; of those, 19 drugs (61.3%) had a survival benefit of less than 3 months, and 30 drugs (96.8%) had an OS improvement of less than 6 months. Of the 12 of 31 drugs (38.7%) with survival increases of more than 3 months, only 1 drug improved absolute survival by more than 6 months. This drug, olaratumab, was granted accelerated approval with orphan status as a treatment for sarcoma in 2016.

Treatment outcomes for PFS were also consistent across subsets for solid tumor (HR, 0.53; 95% CI, 0.48-0.58), nonorphan (HR, 0.59; 95% CI, 0.54-0.66), accelerated approval (HR, 0.54; 95% CI, 0.44-0.67), and full nonaccelerated approval (HR, 0.51; 95% CI, 0.46-0.57) indications (Table 3). Treatment outcomes for hematological cancer (HR, 0.43; 95% CI, 0.28-0.66) and orphan (HR, 0.44; 95% CI, 0.38-0.52) indications were slightly higher. Median PFS increases (2.70 months [IQR, 1.61-4.29 months] overall) were similar across all subsets, ranging from 2.07 months (IQR, 1.10-4.11 months) for nonorphan indications to 4.30 months (IQR, 3.95-10.05 months) for hematological cancer indications.

Treatment outcomes for tumor response ranged from an RR of 1.69 (95% CI, 1.10-2.61) for accelerated approval indications to an RR of 2.64 (95% CI, 2.03-3.43) for orphan indications. The relative effect sizes for PFS were a mean of 38% larger (median, 1.38; IQR, 1.06-1.74) than those for OS. Among 65 total RCTs, the HRs for OS were reported for 54 studies (83.1%), and the HRs for PFS were reported for 53 studies (81.5%). For 50 RCTs (76.9%), the HRs for both PFS and OS were available.

The statistical heterogeneity between the clinical trials was moderate for OS (*I*^2^ = 46%) effect size and high for PFS (*I*^2^ = 88%) and tumor response (*I*^2^ = 91%) effect sizes. This between-study heterogeneity was not readily explained by disease type, orphan status, or approval pathway.

The results were consistent in various sensitivity analyses using different meta-analytical approaches and in subset analyses of different tumor types, drug classes, and lines of treatment as well as in subset analyses of only clinical trials with double-blinding and indications requiring biomarkers for FDA approval (eTable 1 and eTable 2 in the Supplement).

## Discussion

Over 17 years, 92 novel therapies for various types of cancer were approved by the FDA on the basis of 127 clinical trials that met our inclusion criteria. The typical cancer drug was approved on the basis of 1 clinical trial. Clinical trials were mostly nonblinded; almost one-half of them were single-arm clinical trials, and 51.2% of them included fewer than 200 participants. The studies typically found favorable treatment outcomes for PFS; however, for OS, the CIs were also frequently compatible with unfavorable outcomes at the 95% CI level. These data available at FDA approval indicate that novel drug treatments are often associated with substantial benefits for tumor response, with favorable HRs for PFS and OS; however, the treatments prolong patients’ median OS by only 2.40 months or 73 days. Of all 31 drugs approved between 2000 and 2016 with information on median OS improvement, 19 drugs (61.3%) had an estimated OS improvement of less than 3 months, and 30 drugs (96.8%) had an OS improvement of less than 6 months.

Drugs approved as treatment for hematological cancers either had orphan status, underwent accelerated approval, or both. The evidence supporting their approval were typically obtained from a relatively small number of patients who were predominantly enrolled in single-arm and phase 2 clinical trials. In contrast, drugs for solid tumors, drugs without orphan indications, and drugs without accelerated approval pathways entered the market with evidence that was more frequently obtained from RCTs and larger patient samples.

Relative treatment outcomes were better for surrogate outcomes (PFS and tumor response) than for OS. There was moderate to high statistical heterogeneity across treatment effect sizes, but the overall range of effect sizes was similar across the various subsets. The only drug that indicated absolute survival increases of more than 6 months was olaratumab. In this case, the clinical trial reported a survival improvement of almost 1 year, while PFS was prolonged by only 2.5 months.^25^ Notably, a confirmatory clinical trial did not substantiate the OS benefit,^26^ and olaratumab was subsequently withdrawn from the market.^27^ This example highlights the relevance of careful considerations of surrogacy issues and the emerging uncertainty that can occur when drugs are approved despite limited evidence. For 2 drugs (ixazomib and ruxolitinib) for which approval evidence was compatible with unfavorable outcomes for PFS and OS, updated results with longer follow-up periods that were published after treatment approval revealed a benefit for PFS^28^ and OS^29^; however, such updated results did not indicate similar benefits for the other drugs (nivolumab and fulvestrant) evaluated,^30,31^ one of which was tested in 2 clinical trials that were considered pivotal.

Our findings are consistent overall with other evaluations of drug approval data from the US and Europe.^16^ In an analysis of 71 approvals granted by the FDA for solid tumor indications between 2002 and 2014, Fojo et al^14^ found a median survival benefit of 2.1 months for OS and 2.5 months for PFS. Salas-Vega et al^4^ analyzed all 62 cancer drug indications approved by the FDA and the European Medicines Agency between 2003 and 2013 for new molecular entities and estimated a mean OS increase of 3.43 months overall and 2.61 months across all hematological cancer indications. Davis et al^32^ reported a median OS increase of 2.7 months (range, 1.0-5.8 months) across 48 cancer drugs approved by the European Medicines Agency between 2009 and 2013. Other researchers have reported that subsequent studies performed after licensing often do not document survival benefits.^10,33^ For example, a study by Gyawali et al^2^ indicated that, after approval, OS benefits were found for only 15 of 93 cancer drugs approved via the FDA accelerated approval pathway. Our findings are consistent with the results of these studies; even if a survival benefit was found for all drugs, the absolute increase in survival time would typically be small despite the substantial improvement in RRs, ORs, and HRs.

### Limitations

This study has several limitations. First, our analysis was restricted to data presented to the FDA and reported in approval packages. There may be other studies that have evaluated the drugs for these indications. We assumed that a manufacturer would present the most favorable supporting evidence to the FDA. Moreover, our sample not only included clinical trials that were explicitly labeled as pivotal, it included any RCT of the same target population. Thus, because it is more likely that positive results were submitted for approval, there is a low risk that the results from the included clinical trials underestimated the actual benefits of the approved drugs.

Second, our analysis was restricted to drugs that received FDA approval and entered the market. This sample is highly selective and, thus is prone to substantial regression to the mean,^34^ which would lead to inflated benefits.

Third, we examined indications for drugs that were approved for the first time. Data regarding further indications for which a drug was subsequently approved were not considered. For some drugs, we are aware of more substantial benefits for OS or PFS that were found in later-approved indications (eg, nivolumab as a first-line treatment for metastatic melanoma was associated with an increase in 5-year survival from 26% to 52%^30,35^),but we are also aware of drugs without such benefits for other indications (eg, ramucirumab^36,37,38,39,40^). Because we only considered data that were available at the time of approval, additional insights from longer follow-up periods were usually not available, and we did not assess, for example, 5-year survival outcomes.

Fourth, we focused on drug indications, clinical and regulatory details, clinical trial characteristics, and reported treatment outcomes but did not conduct an appraisal of the evidence with a thorough risk-of-bias assessment. Naci et al^41^ reported that, for European Medicines Agency approvals, there are substantial concerns about bias in one-half of all randomized approval studies. Although major sources of bias resulting from a lack of blinding or randomization are reflected in our data, we did not perform a detailed assessment of each study. This limitation is important, as suboptimal controls would produce overestimation of the benefits of the experimental drug.^3^ Again, all of these factors may have led us to overestimate the benefits of the evaluated drugs.

Fifth, while OS, PFS, and tumor response are the most frequently used outcomes in clinical trials of cancer treatments,^42^ we did not collect data on quality of life and adverse effects. Given the relatively small observed improvement in survival, the quality of the last days of a patient’s life is certainly important and warrants closer attention in future research.

Sixth, we combined treatment outcomes across a wide range of different tumor types and drug classes. The heterogeneity between studies for HRs and tumor response rates was moderate for OS and high for PFS and RR. However, it is reasonable to pool outcome data, even in the case of high heterogeneity,^43^ and all subset analyses investigating treatment outcomes within different tumor types, drug classes, and lines of treatment revealed no substantial differences for the overall interpretation.

Seventh, our estimate of the median increase in survival was based on RCTs only, which potentially underestimated the actual merits of some treatments that are used in routine care. In our sample, a survival increase of more than 6 months, which occurred for only 1 drug, was notable. Such outcomes may be found in non-RCTs, which we did not evaluate owing to their clear limitations. An empirical evaluation of European Medicines Agency approvals indicated that several, but not all, approvals that were based on nonrandomized data had large effect sizes.^44^ There are also examples of what may be considered revolutionary treatments,^45^ such as imatinib, which underwent accelerated approval in 2001 for the treatment of chronic myeloid leukemia without the use of RCT evidence. Nonrandomized research using historical controls indicated that patients who received imatinib therapy had an almost normal life expectancy. These data suggest that a survival benefit of substantially more than a median of 73 days is possible; however, based on our findings, such exceptions do not represent the reported outcomes for most of the novel drugs approved for the treatment of many cancers.

## Conclusions

Overall, the data from 17 years of studies conducted for the approval of novel cancer drugs indicate that patients and clinicians typically have limited information available when a novel cancer treatment enters the market; data from RCTs are available for only one-half of indications. This lack of data is even more problematic for patients with hematological cancers. Although these novel therapies may have substantial consequences for tumor size or other markers of tumor response, they were associated with prolonging the life of patients by a median of only 73 days. Our findings suggest that these novel drugs should be used cautiously without the expectation that they will markedly extend survival. Moreover, additional clinical trials performed after a drug is licensed can offer insights on the exact benefit the drug may confer. Many of these drugs were approved to address an unmet medical need. We believe this need still exists.

## Notes

Supplement. **eTable 1.** Sensitivity Analyses **eTable 2.** Subgroup Analyses **eFigure 1.** Orphan Status and Approval Pathway of All Novel Cancer Drug Indications Between 2000 and 2016 **eFigure 2.** Forest Plot of All Randomized Clinical Trials With Data on Progression-Free Survival Used for Approval of Novel Cancer Drugs Between 2000 and 2016
