# Supplementary material for: Clinical Trial Evidence Supporting US Food and Drug Administration Approval of Novel Cancer Therapies Between 2000 and 2016
Source: JAMA Netw Open. 2020 Nov 10;3(11):e2024406. doi: 10.1001/jamanetworkopen.2020.24406 (PMC7656288; doi:10.1001/jamanetworkopen.2020.24406)
Supplement: Supplement. — eTable 1. Sensitivity Analyses eTable 2. Subgroup Analyses eFigure 1. Orphan Status and Approval Pathway of All Novel Cancer Drug Indications Between 2000 and 2016 eFigure 2. Forest Plot of All Randomized Clinical Trials With Data on Progression-Free Survival Used for Approval of Novel Cancer Drugs Between 2000 and 2016 [file jamanetwopen-e2024406-s001.pdf]

Supplementary Online Content

Ladanie A, Schmitt AM, Speich B, et al. Clinical trial evidence supporting US Food and Drug Administration approval of novel cancer therapies between 2000 and 2016. *JAMA Netw Open*. 2020;3(11):e2024406. doi:10.1001/jamanetworkopen.2020.24406

eTable 1. Sensitivity Analyses

eTable 2. Subgroup Analyses

eFigure 1. Orphan Status and Approval Pathway of All Novel Cancer Drug Indications Between 2000 and 2016

eFigure 2. Forest Plot of All Randomized Clinical Trials With Data on Progression-Free Survival Used for Approval of Novel Cancer Drugs Between 2000 and 2016

This supplementary material has been provided by the authors to give readers additional information about their work.

© 2020 Ladanie A et al. *JAMA Network Open*.

eTable 1. Sensitivity Analyses

		Overall survival				Progression-free survival				Tumor Response		
		N RCTs	Effect (HR, 95% CI)	Heterogeneity I^2 (%)		N RCTs	Effect (HR, 95% CI)	Heterogeneity I^2 (%)		N RCTs	Effect (RR & OR, 95 CI)	Heterogeneity I^2 (%)
Hartung-Knapp-Siddik-Jonkman method*		54	0.77 (0.73, 0.81)	46		53	0.52 (0.46, 0.58)	88		50	2.37 (1.88, 2.98)<br>3.63 (2.71, 4.86)	91<br>85
Fixed-effect model		54	0.77 (0.74, 0.80)	46		53	0.58 (0.56, 0.60)	88		50	1.74 (1.67, 1.82)<br>2.69 (2.50, 2.90)	91<br>85
No extrapolation of effects*,**		50	0.76 (0.72, 0.80)	45		50	0.52 (0.47, 0.57)	89		-	-	-
Double-blind comparisons only*		27	0.74 (0.70, 0.79)	37.5		25	0.47 (0.41, 0.54)	89		24	2.71 (2.04, 3.59)<br>4.64 (3.02, 7.11)	93<br>88

Summary HR (95% CI) *Random effects meta-analysis. **The 95% confidence interval or HR on OS and PFS was unavailable for four and three RCTs. Abbreviations: CI, confidence interval; HR, hazard ratio; OR, odds ratio; RR, risk ratio; RCT, randomized controlled trial.

The meta-analyses were completed using R version 3.5.1 and the 'meta' package.
Hartung-Knapp-Siddik-Jonkman method: Hartung-Knapp-Sidik-Jonkman adjustment for random effects model, DerSimonian-Laird estimator for tau^2. Fixed-effects model – Fixed effect model for meta-analysis, DerSimonian-Laird estimator for tau^2. No extrapolation of effects, Double-blind comparisons only, Therapies based on predictive markers only* – Meta-analysis using a random effects model, DerSimonian-Laird estimator for tau^2

© 2020 Ladanie A et al. *JAMA Network Open*.

eTable 2. Subgroup Analyses

		Overall Survival				Progression-free Survival		
Subgroups	*N RCT	HR (CI 95%; I²)	*N RCT	Median Gain in months (IQR)	*N RCT	HR (CI 95%; I²)	*N RCT	Median Gain in months (IQR)
Disease Entity								
Breast Cancer	8	0.83 (0.72, 0.96; 47)	6	2.35 (0.69, 3.83)	8	0.83 (0.72, 0.96; 47)	9	2.21 (1.55, 5.00)
Endocrine and Neuroendocrine	3	0.80 (0.64, 1.00; 0)	1	5.68 (5.68, 5.68)	3	0.8 (0.64, 1.00; 0)	2	10.95 (9.07, 12.82)
Gastrointestinal Cancer	8	0.74 (0.67, 0.83; 43)	8	1.62 (1.40, 2.83)	9	0.74 (0.67, 0.83; 43)	9	1.00 (0.30, 2.20)
Genitourinary	9	0.73 (0.67, 0.80; 38)	7	3.60 (2.40, 4.05)	9	0.73 (0.67, 0.80; 38)	9	2.72 (2.00, 4.00)
Gynecologic	1	0.85 (0.48, 1.51; NA)	1	2.70 (2.70, 2.70)	1	0.85 (0.48, 1.51; NA)	1	7.10 (7.10, 7.10)
Lymphoma	3	0.67 (0.40, 1.14; 16)	1	1.10 (1.10, 1.10)	3	0.67 (0.4, 1.14; 16)	2	11.90 (11.9, 11.90)
Multiple Myeloma	3	0.82 (0.70, 0.95; 0)	1	3.20 (3.20, 3.20)	3	0.82 (0.7, 0.95; 0)	4	4.00 (3.62, 4.20)
Neurologic	1	0.58 (0.37, 0.91; NA)	-	n.r.	1	0.58 (0.37, 0.91; NA)	-	n.r.
Other**	4	1.02 (0.82, 1.27; 0)	2	1.92 (0.54, 3.31)	1	1.02 (0.82, 1.27; 0)	-	n.r.
Sarcoma and GIST	3	0.64 (0.41, 1.02; 78)	2	6.20 (3.39, 9.00)	3	0.64 (0.41, 1.02; 78)	3	2.67 (2.58, 3.44)
Skin	6	0.62 (0.50, 0.78; 51)	1	3.68 (3.68, 3.68)	6	0.62 (0.5, 0.78; 51)	6	2.85 (0.97, 3.61)
Thoracic	5	0.84 (0.74, 0.96; 52)	5	1.60 (-0.10, 1.97)	5	0.84 (0.74, 0.96; 52)	5	1.8 (0.47, 2.20)
Drug Class								
Antibody-drug conjugate	2	0.71 (0.55, 0.93; 8)	1	5.80 (5.80, 5.80)	2	0.64 (0.55, 0.76; 0)	2	4.10 (3.65, 4.55)
Cytostatica	9	0.80 (0.71, 0.90; 55)	9	2.40 (1.10, 2.70)	9	0.58 (0.49, 0.70; 83)	9	1.55 (1.40, 2.10)
Hormonal therapy	4	0.83 (0.65, 1.07; 83)	4	3.25 (0.98, 4.53)	3	0.63 (0.42, 0.95; 93)	4	2.02 (1.58, 2.87)

© 2020 Ladanie A et al. *JAMA Network Open*.

Checkpoint Inhibitor	2	0.79 (0.52, 1.20; 61)	1	3.68 (3.68, 3.68)	2	0.66 (0.53, 0.83; 0)	2	0.30 (0.20, 0.40)
Monoclonal antibody	10	0.73 (0.65, 0.82; 41)	6	2.85 (1.45, 4.57)	10	0.55 (0.44, 0.68; 88)	10	3.30 (1.23, 4.45)
Other***	3	0.79 (0.67, 0.92; 31)	2	3.40 (3.30, 3.50)	2	0.72 (0.55, 0.93; 71)	2	4.00 (3.95, 4.05)
Targeted therapy	24	0.76 (0.69, 0.83; 43)	12	1.70 (0.74, 2.92)	24	0.42 (0.36, 0.49; 88)	21	3.30 (2.21, 5.00)
Treatment Line								
1st	25	0.74 (0.67, 0.81; 50)	15	3.60 (1.35, 4.15)	22	0.47 (0.4, 0.56; 88)	20	3.75 (2.40, 5.01)
2nd	25	0.78 (0.72, 0.84; 51)	16	1.88 (0.82, 2.93)	26	0.54 (0.48, 0.61; 87)	26	2.15 (1.10, 3.87)
3rd or beyond	4	0.81 (0.73, 0.91; 0)	4	2.70 (2.37, 2.83)	4	0.52 (0.35, 0.76; 92)	4	2.70 (1.20, 4.70)
Therapies based on Predictive Markers Only	15	0.79 (0.68, 0.93; 68)	9	0.30 (-0.10, 2.70)	15	0.51 (0.42, 0.63; 89)	15	3.30 (2.20, 5.00)

* Not all data were reported for all trials. ** Includes myelodysplastic syndromes (n = 2), myelofibrosis and multicentric Castleman's disease. ***Includes bortezomib, ixazomib, panobinostat, radium-223 dichloride: Abbreviations: CI, confidence interval; HR, hazard ratio;IQR, interquartile range; RCT, randomized controlled trial; n.r., not reported.

© 2020 Ladanie A et al. *JAMA Network Open*.

eFigure 1. Orphan Status and Approval Pathway of All Novel Cancer Drug Indications Between 2000 and 2016

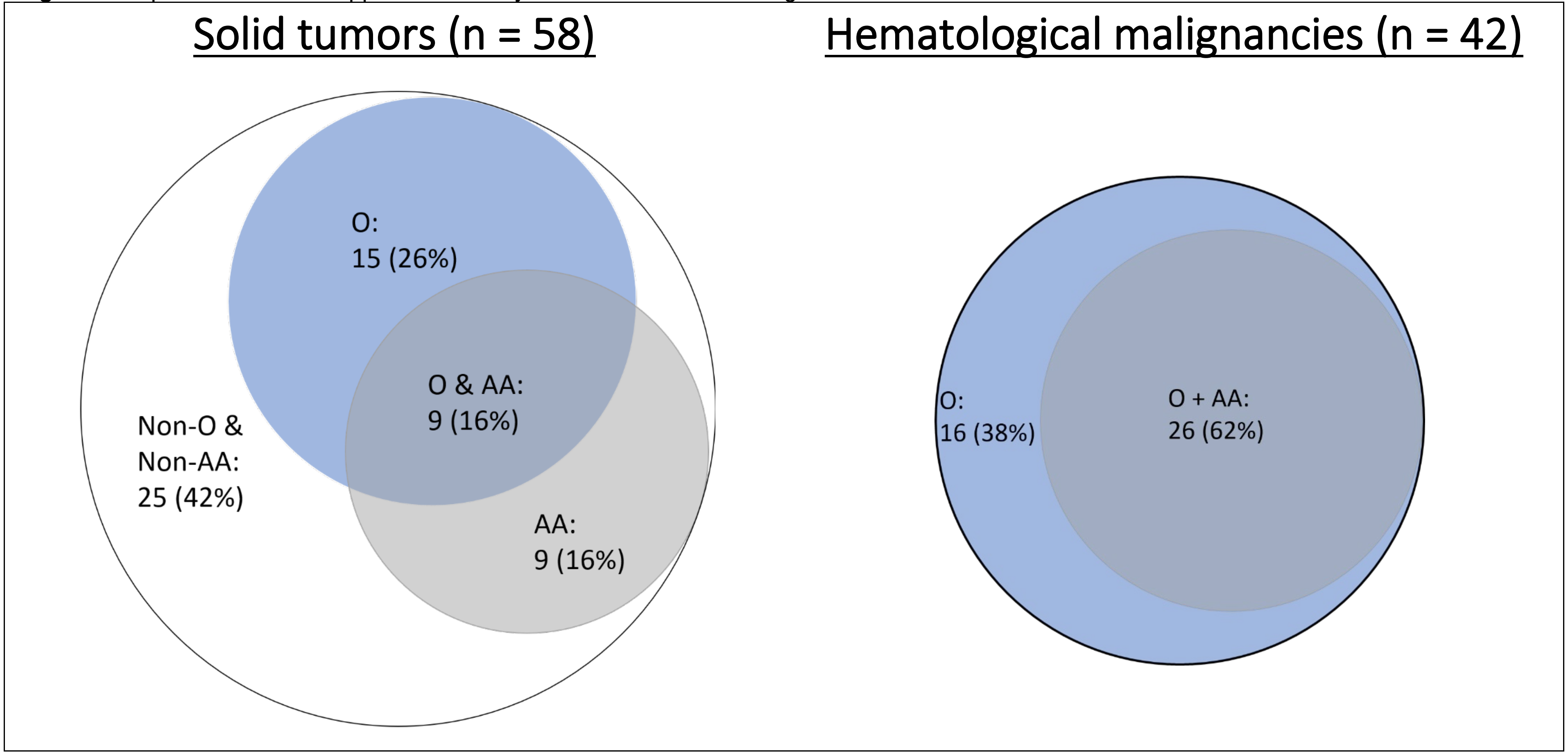

Venn diagram showing proportions of indications with orphan status and accelerated approval and their overlap. Color legend: blue, orphan indication (O); grey, accelerated approved indications (AA); white, standard approval without orphan indications. Left: Of 58 approvals for solid tumors, 25 were without orphan status and accelerated approval. 15 had orphan status alone, 9 received accelerated approval alone, and 9 had both an orphan status and were accelerated approved. Right: There were no approvals without orphan status and without accelerated approval (Non-O & Non-AA). All 42 approvals for hematologic malignancies had orphan status, and the majority (26) also received accelerated approval. Abbreviations: AA, accelerated approval; O, orphan indication; Non-AA, traditional approval (not accelerated approval); Non-O, non-orphan indication.

© 2020 Ladanie A et al. *JAMA Network Open*.

eFigure 2. Forest Plot of All Randomized Clinical Trials With Data on Progression-Free Survival Used for Approval of Novel Cancer Drugs Between 2000 and 2016

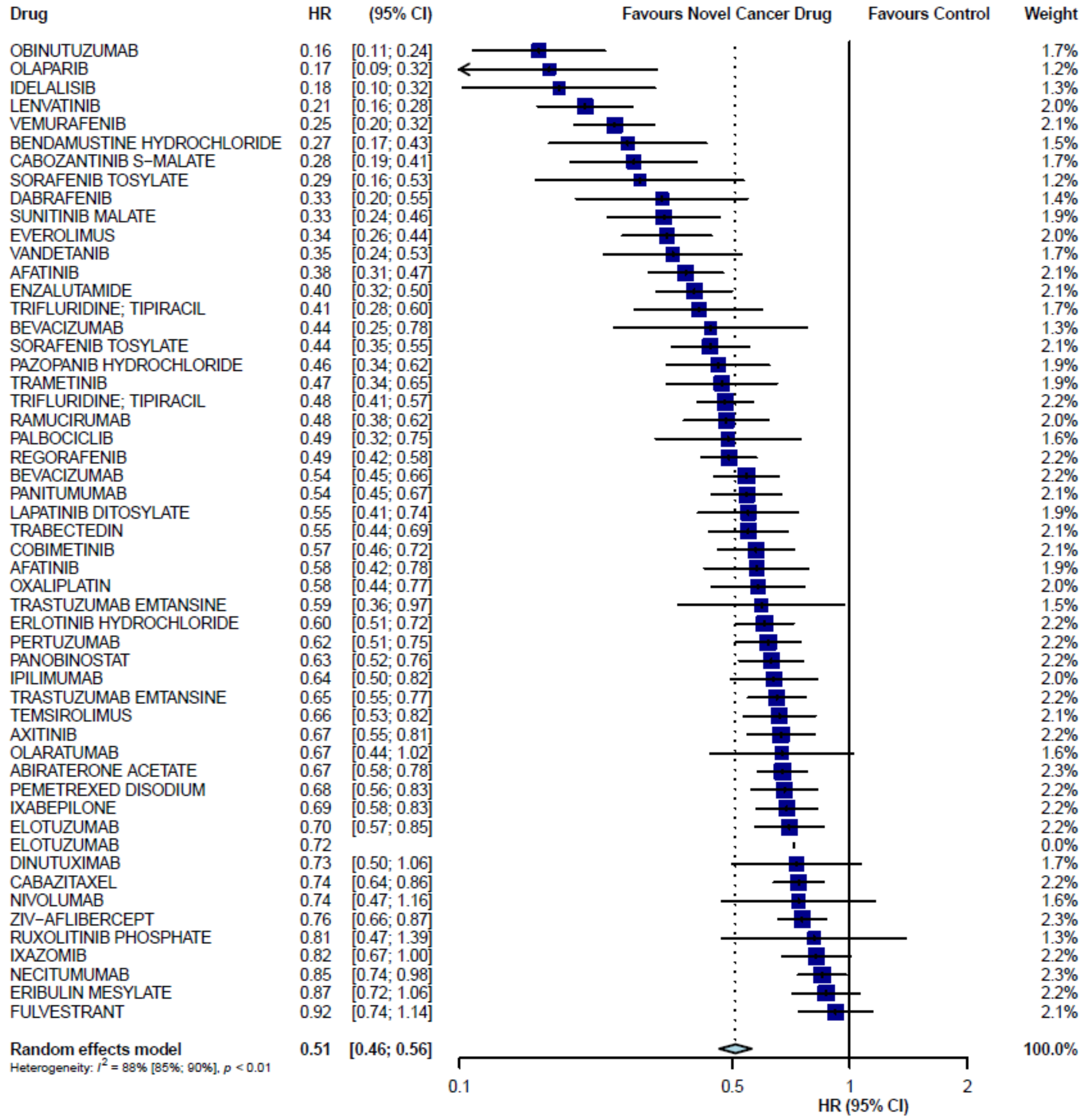

© 2020 Ladanie A et al. *JAMA Network Open*.
